# The Association between Parental Marital Satisfaction and Adolescent Prosocial Behavior in China: A Moderated Serial Mediation Model

**DOI:** 10.3390/ijerph19095630

**Published:** 2022-05-05

**Authors:** Ruiping Zhang, Yaqian Guo, Baoyu Bai, Yabing Wang, Linlin Gao, Lan Cheng

**Affiliations:** 1 Department of Psychology, School of Education, Zhengzhou University, Zhengzhou 450001, China; flyrui@126.com (R.Z.); gyq20210528@163.com (Y.G.); wangyabing012@163.com (Y.W.); 18336461425@163.com (L.G.); chengshanfeng2021@163.com (L.C.); 2 Department of Psychology, School of Philosophy, Wuhan University, Wuhan 430072, China

**Keywords:** parental marital satisfaction, parent–child relationship, empathy, prosocial behavior

## Abstract

Parental marital satisfaction is a well-established protective factor for prosocial behavior in adolescents, yet the parental socialization of adolescent prosocial behavior is limited in Chinese culture. Furthermore, it remains unknown whether the association between parental marital satisfaction and adolescent prosocial behavior is mediated by parent–child intimacy and/or adolescent empathy. The present study examined these associations in 480 students (50.4% male, age = 12.7 ± 0.54 years) and one of their parents. Path analysis (Mplus) revealed that parental marital satisfaction was related to a high level of parent–child intimacy, which was, in turn, associated with a high level of adolescent empathy, which itself predicted adolescent prosocial behavior. The serial mediation effect differed between boys and girls. Specifically, compared with boys, girls’ parent–child intimacy was a stronger predictor of their empathy, and empathy had a stronger predictive effect on their prosocial behavior. Several implications for interventions and policies for increasing prosocial behavior in children are suggested.

## 1. Introduction

Among other behaviors that are conducive to social harmony, prosocial behavior is an individual’s behavioral tendency to help, cooperate, share, and provide comfort during social interactions [1]. It predicts better social adaptation in adolescents and better interpersonal relationships in childhood and adolescence, especially in cultures emphasizing collectivistic orientations (e.g., China) [2]. Prosocial behaviors may be particularly adaptive in facilitating positive development in adolescents among cultures with a collectivistic orientation (e.g., Chinese culture). Given the value of prosocial behavior, it is important to consider the socialization processes that may contribute to these behaviors. Identification of the factors contributing to prosocial behavior in adolescents is the first step toward developing approaches aiming to encourage more prosocial behavior.

The factors that influence adolescent prosocial behavior can broadly be grouped into the following categories: individual, family, social, and situational [2,3]. Parents have been consistently identified as principal socializers in promoting adolescent prosocial behavior [1,3], and there are multiple pathways, including warm parenting, through which this may occur. Previous studies have emphasized the role of parenting behaviors (i.e., authoritative parenting, acceptance, or harsh parenting) in fostering prosocial development in youth [4,5]. In addition, evidence has recently accumulated to suggest that parental marital quality is a strong predictor of prosocial behavior [6,7]. Marital quality is the key antecedent of parenting practice, which ultimately contributes to children’s socialization outcomes. More specifically, Lindsey, Colwell, Frabutt, and MacKinnon-Lewis (2006) [8] have identified that spousal harmony is positively correlated with children’s prosocial behavior. Through socialization processes, parents exert a significant influence on the attitudes and behaviors that affect adolescent prosocial behavior [4].

Despite accumulating studies regarding the parental socialization of adolescent prosocial behavior, research in non-Western countries is scarce, and only limited research has examined the role of parental marital quality in this process. To date, only one study has shown that parental marital quality is a significant predictor of Chinese adolescent prosocial behavior [6]. In the present study, we examine the relationship between parental marital quality and adolescent prosocial behavior to better understand this important phenomenon.

### 1.1. Marital Satisfaction and Adolescents’ Prosocial Behavior

Marital quality, reflected in factors such as levels of marital satisfaction and marital conflict, has long been considered to play a crucial role in the emotional and relational structure of the whole family [9], and it is the core factor that affects child development [10]. According to Bowen’s family system theory, the marital quality of the husband-and-wife system exerts an important influence on the parent–child system and the child’s social adaptation [11]. Children whose parents have poor marital quality have been found to exhibit more internalizing and externalizing problems, poorer health, and lower-quality home environments [12].

Previous studies have mainly focused on marital conflict, which is significantly associated with children’s prosocial behavior. For example, McCoy et al. (2009) [7] found that marital conflict was a significant predictor of prosocial behavior in children ranging from five to seven years of age. Although when entering the adolescent period, individuals tend to be more involved in peer communication, family factors still remain important in affecting their emotional and behavioral development [13,14]. Similarly, Hart and Carlo (2005) [15] have noted important developmental changes in prosocial behavior during this age period.

Importantly, there is increasing evidence that a couple’s capabilities to compensate for negativity with positivity may be a stronger predictor of marital outcomes [16] and parental marital satisfaction seems to be a better predictor of adolescent prosocial behavior. Thus, we examined the relationship between parental marital satisfaction and adolescent prosocial behavior in Chinese middle school students. This is important as the mechanisms underlying the link between parental marital satisfaction and adolescent prosocial behavior have not yet been elucidated.

### 1.2. Parent–Child Relationship as a Potential Mediator

According to the family system theory, the family is a micro-system that strongly affects individual development [17], and the parent–child relationship is a key factor influencing child development and adaptation [18]. According to the spillover hypothesis, feelings, emotions, and behavior in the marital relationship are likely to spill over into the parent–child relationship [19]. Liang et al. (2013) [20] further found that parent–child intimacy, but not parent–child conflict, was a significant predictor of parental marital satisfaction. Therefore, we considered intimacy as one of two core dimensions of the parent–child relationship.

There are good reasons to think that marital satisfaction is connected with child prosocial behavior. For example, Lindsey et al. (2006) [8] found that children’s prosocial actions have been shown to correlate positively with spousal harmony. In addition to marital quality directly influencing children’s behavior, the marital relationship may influence children’s behavior through how a parent feels and acts towards their child [21]. Moreover, Chen, Yuan, Wang, and Zhang (2018) [22] found that parental conflict was negatively associated with children’s problem behaviors through the parent–child relationship in a Chinese sample. In the present study, we examined whether parental marital satisfaction was associated with adolescent prosocial behaviors through the parent–child relationship.

### 1.3. Empathy as Another Potential Mediator

Empathy is typically defined as one’s capacity to understand another person’s perspectives and experience affective responses to another person’s emotional state or condition [1]. For children, developing the ability to show empathy for others is essential for successful interpersonal and social adaptation because it plays an important role in social functioning and social competencies throughout life [23]. According to the empathy–altruism hypothesis [24], a person with high levels of empathy cares more about others’ feelings, which can induce altruistic motives and prompt engagement in more prosocial behavior. Consistent with this hypothesis, numerous studies have shown that empathy is essential for promoting prosocial behavior in children [25]. In terms of potential predictors of prosocial behavior, empathy is considered to be the motivation behind helping behaviors [26].

However, empathy may be impaired by poor marital status in parents [27]. This view fits comfortably with attachment theory [28], which proposes that a secure attachment in the parent–child relationship is an important factor for empathic development in adolescence [12]. Nevertheless, marital conflict often compromises individuals’ attachment security, leading to individuals having difficulties in showing empathic responses to others [29]. Although no research to date has directly examined the mediating role of empathy in the relationship between marital satisfaction and prosocial behavior, Deng et al. (2018) [30] found that the family environment predicted the helping behavior of Chinese adolescents via empathy. Building on the above theoretical and empirical research, we predicted that adolescent empathy mediates the relationship between parental marital satisfaction and adolescent prosocial behaviors.

### 1.4. The Relationship between the Parent–Child Relationship and Empathy

The present study examined the mediating roles of the parent–child relationship and empathy in the relationship between parental marital satisfaction and prosocial behavior. According to Hayes (2013)’s [31], an integrated multi-mediating model can simultaneously explore multiple mechanisms of parental marital satisfaction and prosocial behavior, compared to a simple mediation model. Therefore, the current study provides a deep understanding of the relationship between parental marital satisfaction and adolescent prosocial behavior, which is critical to both the refinement of the theory and to efforts aiming to encourage greater prosocial behavior in adolescents.

However, because of the scarcity of empirical studies, we know little about how these two mediators work together. According to Hayes (2013) [31], there are three possible mediation models: parallel, sequential, and mixed mediation. The large empirical literature shows that parent–child intimacy is related to high levels of empathy [12,32,33,34]. Therefore, parent–child intimacy and empathy may mediate the link between parental marital satisfaction and prosocial behavior in a parallel or/and serial fashion. Due to the exploratory nature of this study, we did not propose a specific hypothesis about how these two mediators would work together. Instead, we utilized a multiple mediation model and postulated our hypothesis as follows: Parent–child intimacy and empathy will play parallel and sequential mediating roles between parental marital satisfaction and prosocial behavior.

### 1.5. The Potential Moderating Effect of Gender

According to ecosystem theory, individuals and their environment will interact with each other and in doing so, affect individual development, and individuals living in the same environment will demonstrate different behaviors due to their different characteristics. In the context of this study, gender is a potentially important moderating variable to be considered in the serial mediation model.

In line with this theory, two meta-analyses have indicated that the association between empathy and prosocial behavior in children varies across gender in both Western [35] and non-Western cultures [36]. However, conflicting results concerning gender-related differences in empathy and prosocial behavior have been reported. For example, Longobardi, Spataro, and Rossi-Arnaud (2019) [37] found significant gender differences in empathic concern and prosocial behavior, with girls outperforming boys in Southern Italy. In a longitudinal study, Van der Graaff, Carlo, Crocetti, Koot, and Branje (2018) [38] found that empathy was more strongly associated with prosocial behavior in adolescent girls than in adolescent boys in the Netherlands. In contrast, the positive effects of empathic concern on children’s prosocial behavior have been found to be stronger in boys than in girls in Northern Italy [39].

Parental parenting practices for children are often shaped by the values and norms of their culture, thereby creating variability in parenting and, ultimately, children’s development [40]. Chinese culture may exacerbate gender-related socialization expectations because boys are raised in a culture of independence and bravery while girls are considered to be kind and considerate. Thus, the socialization of girls is more oriented towards aid and cooperation and is more prosocial and empathic [41]. There is evidence that girls are also more likely to be actively affected by the family environment and thus show more prosocial behavior, compared with boys [38].

Finally, in a sample of Chinese junior high school students, Li et al. (2020) [42] found that the parent–child relationship was associated with adolescent cyberbullying through forgiveness, and gender moderated the mediating effect of forgiveness. Thus, based on these previous findings, we hypothesized that the serial mediation model may differ between boys and girls.

### 1.6. The Present Study

**Figure 1:**
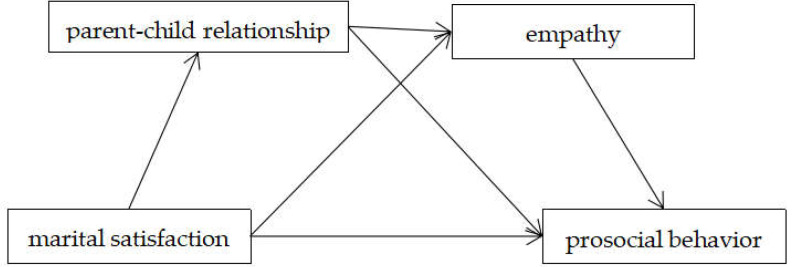
Conceptual model.

This study investigated the predictors of adolescent prosocial behavior in two novel ways: (1) by examining the potential process variables (i.e., mediators) of the relationship between marital satisfaction and adolescent prosocial behavior, and (2) by examining whether gender moderated the above serial mediation model. To our knowledge, no studies have investigated whether the parent–child relationship and empathy can both simultaneously and sequentially mediate the link between parental marital satisfaction and adolescent prosocial behavior. The conceptual framework underpinning this study is shown in Figure 1. We made the following hypotheses:

**Hypothesis** **1** **(H1).** *Marital satisfaction is positively associated with adolescent prosocial behavior*.

**Hypothesis** **2** **(H2).** *The parent–child relationship mediates the relationship between marital satisfaction and adolescent prosocial behavior*.

**Hypothesis** **3** **(H3).** *Adolescent empathy mediates the relationship between parental marital satisfaction and adolescent prosocial behavior*.

**Hypothesis** **4** **(H4).** *The parent–child relationship and adolescent empathy co-play serial mediating roles in the relationship between parental marital satisfaction and adolescent prosocial behavior*.

**Hypothesis** **5** **(H5).** *The serial mediation model differs between genders*.

## 2. Methods

### 2.1. Participants and Procedure

The cluster sampling technique was used with the class as the sampling unit. Participants were 480 adolescents (50.4% male, 46.5% female, and missing 3.1%) and one of their parents, who completed the questionnaires. Adolescents were grade 7 students (age = 12.7 ± 0.54 years), recruited from four middle schools in Henan province. Henan Province is located in the central Plains of China, which is the first household registration of Chinese civilization and also the source and backbone of traditional Chinese culture. Thus, Henan is a typical province and representative of Chinese culture.

Informed consent was obtained from all adolescents and their parents before starting this study. Participants were informed that they could stop the survey at any time if they felt uncomfortable with the questions. Students completed the Interpersonal Reactivity Index [43] and Adolescents’ Prosocial Behavior Scale [44]. Each child was given a parent questionnaire to take home, and one of their parents was asked to complete it. This parent questionnaire included the Relationship Assessment Scale [45] and Child–Parent Relationship Scale [46]. All parent questionnaires were returned. The parent sample included more mothers (*n* = 269, 56%, 43.73 ± 6.16) than fathers (*n* = 203, 42.3%, 44.65 ± 5.67), and the gender of eight parents was not specified (1.7%). About half (50.2%) of parents reported their occupation as being farmworkers, 31.5% working in enterprise and as public officials, 11.3% were self-employed, and the remaining 7% reported their occupation as “other.” With regards to parents’ educational attainment, 56.8% of mothers and 58.5% of fathers had a high school education or lower, and 43.2% of mothers and 41.5% of fathers had a college degree or above. With regard to household yearly income, 27.3% of parents reported this to be less than 10,000 RMB, 51.6% reported this to be between 100,000 and 500,000 RMB, and 21.1% reported this to be above 500,000 RMB.

Most adolescents were native Han (96%), lived with both parents (85.6%), and came from families with two children (93.3%). The research protocol was approved by the Zhengzhou university’s research ethics committee (Ethics number: 202201).

### 2.2. Measures

#### 2.2.1. Marital Satisfaction

The Relationship Assessment Scale (RAS), developed by Hendrick, Dicke, and Hendrick (1998) [45], was used to measure parents’ satisfaction with their marital relationship. The scale has seven items that are scored on a five-point scale ranging from 1 (disagree strongly) to 5 (agree strongly). A sample item is “I wish I hadn’t got married.” The validated Chinese version of the RAS was used in this study [47,48,49]. In the present study, a confirmatory factor analysis revealed that the one-factor model provided a good fit for the data, χ^2^/*df* = 1.728, Tucker–Lewis Index (TLI) = 0.992, comparative fit index (CFI) = 0.996, root mean square error of approximation (RMSEA) = 0.039. The Cronbach’s alpha coefficient in this study was 0.89.

#### 2.2.2. Adolescents’ Prosocial Behavior

Prosocial behavior was assessed with the revised version of the Prosocial Tendencies Measure [44], which was developed by Carlo and Randall (2002) [50]. Kou et al. (2011) [44] revised this scale based on the Chinese cultural background and the developmental situation of adolescence. This questionnaire has 15 items that are rated on a seven-point scale ranging from 1 (strongly disagree) to 7 (strongly agree). A sample item is “I like to participate in social welfare activities organized inside and outside the school.” Higher scores indicate more prosocial behavior. A confirmatory factor analysis showed that the one-factor model provided a good fit for the data, χ^2^/*df* = 1.993, TLI = 0.961, CFI = 0.970, RMSEA = 0.046. The Cronbach’s alpha coefficient in this study was 0.90.

#### 2.2.3. Adolescents’ Empathy

The Interpersonal Reactivity Index Scale was used to measure adolescents’ empathy [43], and the Chinese version of the IRI shows acceptable reliability and validity in Chinese adolescents [51]. The scale includes the four following dimensions: perspective taking, imagination, empathic concern, and personal distress. Children scored the items on a five-point scale ranging from 1 (strongly disagree) to 5 (strongly agree). A sample item is “I often worry about those less fortunate than me.” As per Siu and Shek (2005) [51], the perspective taking and empathy concern subscales were considered most representative of empathy and adopted in this study. In this study, the Cronbach’s alpha coefficient of adolescent empathy was 0.71.

#### 2.2.4. The Parent–Child Relationship

The parent–child relationship was measured by the Chinese version of the Child–Parent Relationship Scale [52], revised by Zhang et al. (2008) [46]. The scale consists of 30 items that span the three dimensions of intimacy, dependence, and conflict to measure the quality of parent–child relationships. The intimacy dimension was selected for this study, which included 10 items. One example item is “I have a close relationship with my children.” Fathers or mothers scored the items on a five-point scale ranging from 1 (completely disagree) to 5 (completely agree). This measure had adequate internal consistency in the present sample (α = 0.81). The validated Chinese version of the parent–child relationship was used in the present study [18].

### 2.3. Data Analysis

SPSS 22.0 (IBM, Chicago, IL, USA) and Mplus 7.0 ( Los Angeles, CA, USA) were used for statistical analysis. First, descriptive statistics and correlation analyses were conducted in SPSS 22.0. All models were adjusted for demographic covariates including, child gender (coded as 0 = boy, 1 = girl), parental educational attainment (i.e., the mean of mother and father’s education; coded as 0 = high school education or below, 1 = college degree or above), and family yearly income (coded from 1 = below 10,000 RMB to 3 = above 50,000 RMB). The predictors were not mean-centered before being entered into all models, except for the moderation analyses when generating the interaction terms.

Then, structural equation modeling was conducted to test the hypothesized model. Maximum likelihood estimation was carried out in Mplus version 7.0. The following goodness of fit indices were used: Chi-square statistic (χ^2^), χ^2^/*df*, the standardized root mean square residual, RMSEA, and CFI. Finally, a multi-group approach was used to test whether gender moderated the serial mediation model. Models in which specific parameters were constrained to be equal across the two gender groups were compared to models in which these parameters were free to vary. The parameter was assumed to differ between boys and girls if the Chi-square result was significantly different between the constrained model and the unconstrained model.

## 3. Results

### 3.1. Preliminary Analyses

Table 1 presents the means and standard deviations of all predictors and outcome variables by gender. The skewness and kurtosis indices showed a normal distribution for all variables (with values between −1 and +1). A multivariate analysis of variance was conducted to compare the mean differences on all the variables. Boys reported to have higher levels of adolescent empathy and prosocial behavior than girls, *F* _(1, 358)_ = 8.572, *p* < 0.01, η_p_^2^ = 0.02; *F* _(1, 436)_ = 17.62, *p* < 0.001, η_p_^2^ = 0.04. The levels of adolescent empathy and prosocial behavior did not differ across family income levels, *F* _(1, 293)_ = 0.18, *p* = 0.84; *F* _(1, 268)_ = 1.02, *p* = 0.36. Adolescents with parents who had higher levels of education attainment reported higher levels of adolescents’ empathy and prosocial behaviors, *F* _(1, 446)_ = 5.13, *p* < 0.05, η_p_^2^ = 0.01; *F* _(1, 365)_ = 5.97, *p* < 0.05, η_p_^2^ = 0.02.

Table 1 also presents presented the zero-order correlations for all predictors and outcome variables by gender. Correlation analyses showed similar results for boys and girls with respect to adolescents’ prosocial behavior; prosocial behavior was positively associated with both the parent–child relationship and adolescents’ empathy. Meanwhile, parental marital satisfaction was positively associated with the parent–child relationship. For both boys and girls, parental marital satisfaction was not significantly associated with adolescent empathy. Despite these similar results, different patterns of correlations were found for boys and girls. For instance, the parent–child relationship was positively associated with adolescent empathy in girls but not in boys. Furthermore, parental marital satisfaction was positively associated with empathy in girls but not boys’ empathy.

**Table 1:** Descriptive statistics and correlations among study variables (*N* = 480).

Variables	Girls (M ± SD)	Boys (M ± SD)	1	2	3	4
1. Marital satisfaction	3.98 ± 0.69	3.98 ± 0.68	-	0.31 **	0.15 *	0.15
2. Parent–child relationship	3.09 ± 0.53	3.05 ± 0.55	0.31 *	-	0.31 **	0.18 **
3. Empathy	3.85 ± 0.62	4.08 ± 0.53	0.08	0.07	-	0.66 **
4. Prosocial behavior	5.33 ± 1.02	5.61 ± 0.83	0.11	0.17 *	0.37 *	-

Correlations for boys are below the diagonal, correlations for girls are above the diagonal. * *p* < 0.05; ** *p* < 0.01.

### 3.2. Mediating Model Analyses

**Figure 2:**
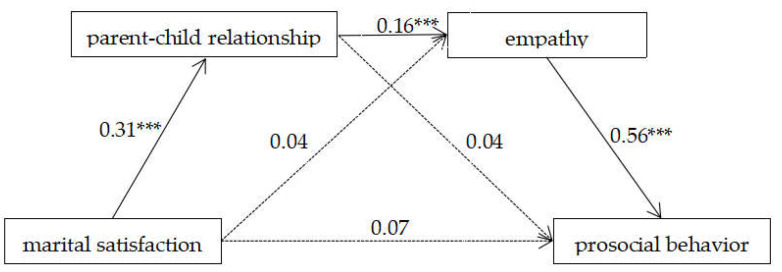
All the path coefficients were standardized; child age and parent education level were controlled for but are not presented in the figure for simplicity; *** *p* < 0.001.

Structural equation modeling was used to test our hypothesized model. Child gender, parent education, and child age were used as control variables in the mediation effect test. The model was saturated. The results of the mediation effect analysis are shown in Figure 2. As expected, marital satisfaction significantly predicted the parent–child relationship (β = 0.31, *p* < 0.001, 95% confidence interval [CI] = [0.22, 0.39]), the parent–child relationship significantly predicted adolescents’ empathy (β = 0.16, *p* = 0.001, 95% CI = [0.06, 0.26]), and adolescent empathy significantly predicted adolescent prosocial behavior (β = 0.56, *p* < 0.001, 95% CI = [0.47, 0.66]). Meanwhile, there were three non-significant pathways from marital satisfaction to adolescents’ empathy (β = 0.04, *p* = 0.38), from marital satisfaction to prosocial behavior (β = 0.07, *p* = 0.14), and from the parent–child relationship to adolescents’ prosocial behavior (β = 0.04, *p* = 0.40). Moreover, the parent–child relationship and adolescents’ empathy mediated the link between marital satisfaction and adolescents’ prosocial behavior, which contained the following significant mediating pathway: marital satisfaction → parent–child relationship → empathy → prosocial behavior (indirect effect = 0.03, SE = 0.01, *p* = 0.01, 95% CI = [0.01, 0.05]).

### 3.3. Multiple-Group Analysis

**Figure 3:**
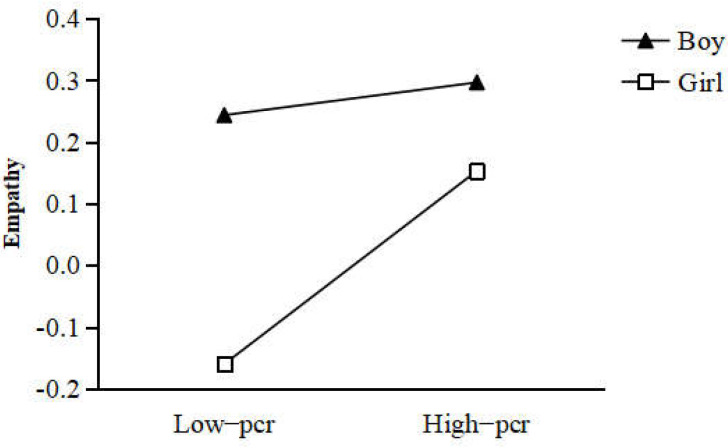
The moderating effect of gender on the relationship between the parent–child relationship and empathy. Note. pcr = parent−child relationship.

**Figure 4:**
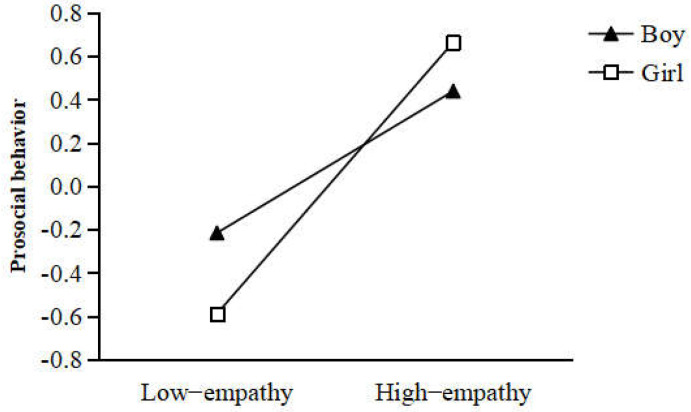
The moderating effect of gender on the relationship between empathy and prosocial behavior.

Multi-group analysis was conducted to test whether the serial mediation model was moderated by gender. The unconstrained model (in which all parameters were allowed to vary freely across boys and girls) and constrained model (in which all parameters were constrained to be equal across boys and girls) were compared to assess whether the serial mediation model was moderated by gender. The results showed that the two models were significantly different in two paths (parent–child relationship → empathy, Δχ^2^ (1) = 5.00, *p* = 0.03; empathy → prosocial behavior, Δχ^2^ (1) = 6.20, *p* = 0.01). These findings indicated that gender moderated the serial mediation model. Specifically, gender moderated the effect of the parent–child relationship on empathy (see Figure 3), as well as the effect of empathy on prosocial behavior (see Figure 4).

## 4. Discussion

This study examined the mediators in the relationship between parental marital satisfaction and adolescent prosocial behavior in Chinese middle school students. Path analysis was used to analyze data from a sample of Chinese adolescents and their parents, and the findings partially supported the hypothesized model. As expected, the results indicated that the relationship between parental marital satisfaction and adolescent prosocial behavior was serially mediated by the parent–child relationship and adolescent empathy. More importantly, the present study revealed the pathways by which parental marital satisfaction was associated with adolescent prosocial behavior, that is, specifically via the sequential mediators of the parent–child relationship and adolescent empathy. In addition, the serial mediation model was found to differ across gender. As such, these results advance our understanding of the association between parental marital satisfaction and adolescent prosocial behaviors.

Inconsistent with our hypothesis, the direct effect of parental marital satisfaction on adolescent prosocial behavior was non-significant, and the parent–child relationship did not mediate the relationship between parental marital satisfaction and adolescent prosocial behavior. Similarly, empathy did not mediate the relationship between parental marital satisfaction and adolescent prosocial behavior. A possible explanation for these findings given by the person–context interaction theory is that situations stimulate the individual’s mood emotion system first and then produce behavior. In other words, the relationship between parental marital satisfaction and individual development was not direct but indirect, arising through individuals and their environments. Although prior studies have investigated the effects of parent–child and empathy on prosocial behavior [12,23], few studies, if any, have examined these two constructs together. As such, this integrated, multiple mediation model provides us with a more comprehensive process accounting for how parental marital satisfaction is related to adolescent prosocial behavior in Chinese culture.

It is noteworthy that the results revealed significant predictive effects of gender and parent education level on adolescent empathy and prosocial behavior, reiterating the crucial role of parents in this cultural socialization process. Specifically, boys scored significantly higher than girls on reported adolescent empathy and prosocial behavior in this study, which is consistent with previous studies [11,53]. Parent education level was positively predictive of adolescent empathy and prosocial behavior, such that adolescents with parents with higher education attainment showed higher levels of prosocial behavior, which comply with the traditional Confucian virtues in the Chinese collective culture.

Moreover, the multiple-group analysis revealed that the serial mediation model differed between boys and girls. Specifically, the parent–child relationship was a predictor of empathy for girls, but not for boys. That is, adolescent girls are greatly influenced by the positive nature of parent–child intimacy [54]. As noted in the Introduction, Chinese parents may exacerbate gender-related socialization expectations because boys are raised in a culture of independence and bravery, while girls are considered to be kind and considerate. Adolescent boys are more eager for independence and tend to perceive their mother’s care as control [55]. This could explain why, during adolescence, a differential relationship between parent–child intimacy and child empathy for boys and girls was found in this study.

The results also revealed that the relationship between empathy and prosocial behavior differed across gender, i.e., the relationship between empathy and prosocial behavior was stronger in girls than in boys. This is inconsistent with previous findings that empathy is not strongly linked to prosocial behavior in girls [37,38]. There is a potential explanation for this finding in gender socialization, which gives meaning to the biological sex differences associated with puberty. Specifically, more developed bodies amplify gender awareness among adolescents, abruptly orienting them to traditional gender norms [56]. In addition, a growing body of literature highlights that the difference may be more due to social culture rather than physical factors [4,5]. In other words, puberty-linked social pressures may lead to a variety of cognitive and behavioral divergences between boys and girls. This means that the relationship between empathy and prosocial behavior may be stronger in girls than in boys, and this may depend on gender stereotypical socialization.

When considering these findings, it is important to note the limitations of the study. First, we used a cross-sectional design that precludes the inference of causality. Second, the limited generalizability of our findings should be acknowledged, and a more diverse range of participants (e.g., different ages, levels of education, areas, and religious backgrounds) is required in future research. Third, although this study demonstrated that marital satisfaction can predict adolescent prosocial behavior through the parent–child relationship and adolescent empathy, the relationship between the parent–child relationship and adolescent empathy may be reciprocal; that is, adolescent empathy may impact the quality of parent–child relationships. Following Hayes’ (2013) [31] suggestion, we took into consideration the possibility of an indirect link from parental marital satisfaction to prosocial behavior through empathy and the parent–child relationship, after gender, age, and parent education had been controlled for. In the reversed serial mediation model, empathy and the parent–child relationship did not mediate the link between parent marital satisfaction and adolescents’ prosocial behavior (indirect effect = 0.008, p = 0.124). In that case, we may see a path from the parent–child relationship to empathy. Consistent with this, Hastings et al. (2015) [57] offers the view that children’s behaviors both shape and are shaped by context. Thus, another assumption should be considered, the fact that there was an indirect link between adolescent prosocial behavior to parental marital satisfaction through parent–child relationship and adolescent empathy, controlling for gender, age, and parent education. In the reversed serial mediation model, the results indicated that the effect of adolescent prosocial behavior on parental marital satisfaction was not serially mediated by the parent–child relationship and adolescent empathy (indirect effect = 0.00, *p* = 0.665). Nonetheless, the cross-sectional nature of this study is still a major limitation. A carefully designed longitudinal study should be conducted to determine the causal relationship between these variables. Future research may consider exploring other types of parental socialization (e.g., parental goals) that may have an impact on adolescent prosocial behavior, as well as other possible mediators linking the associations between parental marital satisfaction and adolescent prosocial behaviors (e.g., parent–child interaction, perspective taking).

Despite these limitations, the serial mediation model offers new insights by revealing the possibilities of other pathways in explaining the relationship between parental marital satisfaction and adolescent prosocial behavior. This study demonstrated the importance of parental marital satisfaction in the improvement of adolescent prosocial behavior in China. These findings also indicate that parents have a better relationship with their children when the relationship with their spouse is satisfactory. Efforts designed to teach parents how to build good relationships with their children may help their children to develop empathy and further promote their prosocial behavior. Our findings also indicated stronger associations between empathy and prosocial behavior and of the parent–child relationship with empathy in girls than in boys, which emphasizes the importance of providing parent–child relationship and empathy prevention and intervention programs for girls to increase their prosocial behavior. There is no denying that sometimes very satisfied spouses forget about their parenting and are only busy with each other. This is the so-called marital type of family, as opposed to the child-centered one. However, according to the current research, in China’s families, which prioritize others’ needs over individual interests, the status of children in the family is very high, and thus, the marital type of families is unlikely to apply.

## 5. Conclusions

The present study yields evidence suggesting that parent–child relationships and empathy mediate the relationship between parental marital satisfaction and prosocial behavior sequentially but not in parallel in a Chinese adolescent sample. In addition, the serial mediation effect differed between boys and girls. Findings provide us with a better understanding of how parental marital satisfaction is related to prosocial behavior, which is conducive to improving adolescent prosocial behavior.

## Data Availability

The data presented in this study are available upon request from the corresponding author.
